# Pyo-pneumothorax tuberculeux: à propos de 18 cas

**DOI:** 10.11604/pamj.2016.24.26.8675

**Published:** 2016-05-09

**Authors:** Souhi Hicham, El Ouazzani Hanane, Janah Hicham, Rhorfi Ismaïl, Abid Ahmed

**Affiliations:** 1 Service de Pneumologie de l'Hôpital Militaire d'Instruction Mohammed V, Rabat, Maroc

**Keywords:** Pyothorax, pneumothorax, tuberculose, Pyothorax, pneumothorax, tuberculosis

## Abstract

Le pyo-pneumothorax tuberculeux est une complication rare mais grave de la tuberculose pulmonaire évolutive. Nous rapportons une série de 18 cas de pyo-pneumothorax tuberculeux colligés au service de Pneumo-Phtisiologie de l'Hôpital Militaire d'Instruction Mohammed V de Rabat entre janvier 2005 et décembre 2009. Il s'agit de 15 hommes et 3 femmes d’âge moyen de 35 ans ±7 ans. 4 patients étaient diabétiques. Le tabagisme était retrouvé chez 9 cas. Le pyo-pneumothorax était du coté droit dans 13 cas. La radiographie thoracique avait montré des lésions cavitaires chez 15 patients et des lésions étendues et bilatérales chez 8 cas. La recherche de BK dans le liquide de tubage gastrique était positive chez 16 cas. Un drainage thoracique associé à un traitement antituberculeux selon le régime 2SRHZ/7RH et une kinésithérapie respiratoire ont été instaurés chez tous les cas. La durée moyenne de drainage pleural était de 4 semaines. Chez 3 cas on avait noté la persistance de la suppuration pleurale ayant nécessité une toilette pleurale sous thoracoscopie avec pleurectomie et exérèse pulmonaire limitée emportant la lésion parenchymateuse tuberculeuse et la persistance d'une volumineuse poche pleurale avec trouble ventilatoire restrictif ayant nécessité une décortication pleurale chirurgicale chez deux cas. L’évolution était favorable avec pachypleurite séquellaire minime chez le reste des cas. Le pyo-pneumothorax tuberculeux est une forme grave, qui est souvent en rapport avec une tuberculose cavitaire active. L’évolution est généralement trainante malgré le traitement antituberculeux et le drainage thoracique, d'où la nécessité d'un diagnostic et un traitement précoce de toute forme de tuberculose.

## Abstract (en)

Tuberculous pyopneumothorax is a rare but serious complication of evolutive pulmonary tuberculosis. We report a series of 18 cases with tuberculous pyopneumothorax admitted to the Pneumo-Phthisiology Department of the Mohammed V Military Teaching Hospital in Rabat between January 2005 and December 2009. Our study included 15 men and 3 women, the average age was 35 ± 7 years. 4 patients were diabetic. Smoking was found in 9 cases. Right-sided pneumothorax was found in 13 cases. Chest radiograph showed cavitary lesions in 15 patients and extensive bilateral lesions in 8 cases. The search for Mycobacterium tuberculosis in the fluid from the gastric tube was positive in 16 cases. Chest drainage associated with antituberculosis treatment according to the 2SRHZ/7RH regimen and respiratory kinesitherapy were performed in all cases. The average duration of pleural drainage was 4 weeks. In 3 cases we noted persistent pleural suppuration requiring pleural toilet using thoracoscopy with pleurectomy and limited pulmonary resection to eliminate tuberculous parenchymal lesions and the persistence of a large pleural pocket with restrictive ventilatory defect that required surgery for pleural decortication in two cases. The outcome was favorable with minimal pachypleuritis as sequelae in the remaining cases. Tuberculous pyopneumothorax is a severe form, which is often associated with active cavitary tuberculosis. Evolution is generally progressive despite antituberculosis treatment and thoracic drainage, hence the need for early diagnosis and treatment of all forms of tuberculosis.

## Introduction

L a tuberculose est un enjeu de santé publique dans le monde et reste une priorité au Maroc où elle peut évoluer vers des formes graves comme pyo-pneumothorax tuberculeux, celui-ci complique le plus souvent une tuberculose pulmonaire cavitaire. Cette pathologie pose encore au pneumologue des difficultés diagnostiques et surtout thérapeutiques. Le but de notre étude est de décrire les aspects cliniques, diagnostiques, thérapeutiques et évolutifs des pyo-pneumothorax tuberculeux recensés au service de Pneumo-Phtisiologie de l'Hôpital Militaire d'Instruction Mohammed V de Rabat entre janvier 2005 et décembre 2009.

## Méthodes

Il s'agit d'une étude rétrospective des dossiers médicaux des patients atteints de pyp-pneumothorax tuberculeux colligés au service de Pneumo-Phtisiologie de l'Hôpital Militaire d'Instruction Mohammed V de Rabat entre janvier 2005 et décembre 2009.

## Résultats

**Population à l’étude:** Notre population représente une série hospitalière de 18 patients atteints de pyo-pneumothorax tuberculeux: il s'agit de 15 hommes (83%) et 3 femmes (17%) ayant un âge moyen de 35 ans ± 7 ans. La prévalence est de 3%.

**Antécédents:** Une intoxication tabagique a été retrouvée chez 50% des cas avec une consommation moyenne de 16 paquets/année (PA). Un diabète a été noté chez 22% des patients.

**Présentation clinique:** Le pneumothorax était inaugural chez 73% des patients. Les autres patients étaient porteurs d'une tuberculose pulmonaire active et ont développé leur pneumothorax alors qu'ils étaient sous traitement antituberculeux. Un contexte d'altération de l’état général a été retrouvé chez la majorité des patients (95%). Les signes fonctionnels respiratoires comportaient: une douleur thoracique dans 75% des cas; une toux dans 65% des cas; une dyspnée dans 45% des cas.

**Présentation radiologique:** Dans tous les cas, c'est la radiographie thoracique qui a confirmé l'hydro-pneumothorax. Dans 72% des cas, il s'agissait d'un hydro-pneumothorax droit. L'atteinte parenchymateuse associée était des opacités excavées dans 83% des cas et des infiltrats réticulonodulaires étendus chez le reste des cas.

**Diagnostic bactériologique et anatomopathologique:** La tuberculose était confirmée par l’étude bactériologique des crachats chez 90% et par l’étude anatomopathologique de la plèvre chez 10%.

**Traitement et évolution:** Un drainage thoracique associé à un traitement antituberculeux selon le régime 2SRHZ/7RH et une kinésithérapie respiratoire ont été instaurés chez tous les cas. La durée moyenne de drainage pleural était de 4 semaines. Chez 3 cas on avait noté la persistance de la suppuration pleurale ayant nécessité une toilette pleurale sous thoracoscopie avec pleurectomie et exérèse pulmonaire limitée emportant la lésion parenchymateuse tuberculeuse. Dans deux cas on note la persistance d'une volumineuse poche pleurale avec trouble ventilatoire restrictif ayant nécessité une décortication pleurale chirurgicale. L’évolution était favorable avec pachypleurite minime séquellaire chez le reste des cas.

## Discussion

La tuberculose reste l'une des maladies transmissibles causant le plus de décès dans le monde. En 2013, selon les estimations, 9 millions de personnes l'ont contractée et 1,5 million de personnes en sont décédées [1]. Le pyo-pneumothorax tuberculeux constitue une forme sévère de la tuberculose. C'est une complication de la tuberculose pulmonaire post-primaire. Il survient après rupture de lésions cavitaires périphériques. Dans d'autres cas, il survient après rupture dans la cavité pleurale d'adénopathies paratrachéales ou d'abcès paravertébraux, secondaires à un mal de Pott. La fréquence du pyo-pneumothorax tuberculeux est variable d'une étude à l'autre, mais elle reste relativement faible. Weissberg et al. ont passé en revue les étiologies de 505 pneumothorax secondaires: ils ont constaté 9 cas de tuberculose, soit une fréquence de 1,8%, tandis que les BPCO ont été notées dans 348 cas (69%) [2]. Hassine et al.ont rapporté, parmi 875 cas de tuberculose pulmonaire recensés entre 1990 et 1999, 28 cas d'empyèmes dont 9 cas de pyopneumothorax, soit une fréquence de 1%. Dans notre série, les pyo-pneumothorax tuberculeux représentent 3% des cas de tuberculose hospitalisés dans la même période [3]. Le pneumothorax tuberculeux touche les hommes plus que les femmes; cette prédominance masculine est rapportée par de nombreuses études [4]. L’âge de survenue du pyo-pneumothorax tuberculeux est nettement inférieur à celui du pneumothorax secondaire aux BPCO: 49,5 ans versus 60 ans. Les manifestations cliniques associent, à des degrés divers, un syndrome infectieux, une altération de l’état général, une toux, une expectoration et une dyspnée. La douleur thoracique est le symptôme le plus constant, et l'hémoptysie est très rare. Cependant, dans certains cas, le pyo-pneumothorax tuberculeux est pauci ou asymptomatique [5].

**Figure 1:**
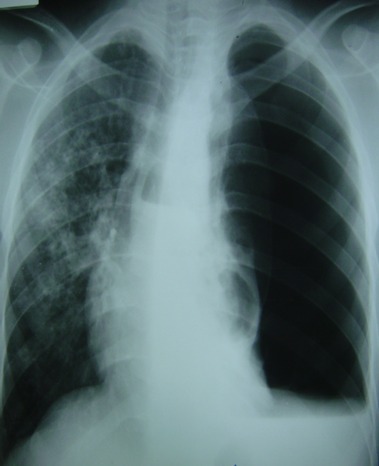
Pyo-pneumothorax gauche associé à un infiltrat excavé controlatéral

La radiographie du thorax standard montre une association de plusieurs images: opacités pleurales enkystées, épaississement pleural, niveau hydro-aérique avec des lésions homolatérales ou bilatérales de tuberculose pulmonaire, et rarement des images de lyse costale. Par ailleurs, la tomodensitométrie thoracique et l'imagerie par résonance magnétique peuvent être utiles pour détecter des lésions nodulaires, une fistulisation cutanée, une ostéite costale ou encore un épaississement pleural. Le diagnostic du pneumothorax tuberculeux est facilement évoqué quand s'associent au pneumothorax des lésions fibro-cavitaires parenchymateuses (Figure 1). Les bacilloscopies positives permettent de confirmer l’étiologie tuberculeuse [6]. Les difficultés se posent quand le pyopneumothorax est isolé: l'isolement du bacille de Koch dans le liquide pleural étant rare la preuve de l'origine tuberculeuse ne peut être parfois apportée que sur pièce de décortication pleurale et/ou d'exérèse pulmonaire. La réaction de polymérisation en chaîne est une technique d'amplification génique d'introduction relativement récente; elle est très sensible pour les échantillons pulmonaires dont l'examen direct est positif, mais montre une grande variabilité pour des échantillons dont l'examen direct est négatif. Dans les cas de pyopneumothorax isolé, elle pourra être réalisée sur des prélèvements de liquide pleural ou sur des fragments biopsiques de la plèvre, mais une réponse négative ne permettra pas d'exclure le diagnostic de tuberculose. La fibroscopie bronchique peut être utile pour le diagnostic d'une fistule bronchopleurale confirmée alors par l'injection de rifampicine intrapleurale, de couleur rouge orangé, qu'on retrouve dans les sécrétions bronchiques et, ensuite, dans les expectorations. La fistule bronchopleurale est fréquemment diagnostiquée sur l'expectoration du pus pleural, aidée ou non par le test d'injection intrapleurale de rifampicine [7].

Le traitement du pyo-pneumothorax tuberculeux repose sur: un traitement antituberculeux correcte; un drainage thoracique parfois guidé par un repérage échographique qui peut permettre la fermeture de la fistule bronchopleurale au bout de quelques semaines pouvant être associé à des lavages-aspirations par du sérum physiologique quotidiens et répétés en utilisant parfois la streptokinase; une kinésithérapie adaptée et bien suivie.

La chirurgie est indiquée pour des situations particulières. Il s'agit alors essentiellement de la décortication pleurale en cas de plèvre épaissie, avec absence de réexpansion du poumon. Des résections pulmonaires (lobectomie ou segmentectomie, voire pneumonectomie) peuvent être nécessaires pour extirper le parenchyme détruit. Une fenêtre ouverte par thoracostomie a été proposée par certains auteurs en cas d'empyème persistant (malgré une thérapeutique bien conduite) associé à une fistule bronchopleurale [7]. Certains auteurs ont suggéré une myoplastie (transposition du muscle pectoral) pour traiter la fistule bronchopleurale. Enfin, la chirurgie thoracique vidéo-assistée a été utile et efficace pour traiter des adhérences pleurales, réaliser des biopsies, voire effectuer des résections pulmonaires de type Wedge [8].

## Conclusion

Le pyo-pneumothorax tuberculeux est souvent en rapport avec une tuberculose cavitaire active, diagnostiquée avec retard. Il s'associe à une lourde morbidité (hospitalisation prolongée, soins médicaux, complications iatrogènes). Son diagnostic est souvent bactériologique et repose sur l'histologie dans les cas difficiles. Le traitement est consensuel, basé sur la chimiothérapie antituberculeuse et le drainage thoracique. L’évolution est souvent favorable, mais des séquelles pleurales à type de pachypleurite plus ou moins importante peuvent persister, occasionnant un retentissement fonctionnel respiratoire. La prévention primaire et le diagnostic précoce de toute tuberculose pulmonaire restent la pierre angulaire de toute stratégie antituberculeuse: elle seule permet d'espérer, dans le futur, une disparition des formes sévères de tuberculose et, notamment, du pyo-pneumothorax tuberculeux.

### Etat des connaissance sur le sujet

- Le pyothorax tuberculeux est une forme grave de la tuberculose nécessitant une prise en charge associant une poly chimiothérapie spécifique, un traitement local et une kinésithérapie.
- Dans certains cas, on peut avoir recours à la chirurgie pour éviter des séquelles handicapantes.

### Contribution de notre étude à la connaissance

- Cette étude met en lumière la présentation clinique et radiologique de cette forme particulière de Tuberculose.
- Par ailleurs, nous rapportons notre expérience pour améliorer sa prise en charge.

## References

[1] OMS (2014). Rapport 2014 sur la lutte contre la tuberculose dans le monde.

[2] Weissberg Dov (2000). FCCP, Yeal Rafaely. Pneumothorax: experience with 1199 patients. Chest..

[3] Hassine E, Marniche K, Bousnina S, Rekhis O, Rabah B, Ben Mustapha MA, Chabbou A, El Gharbi B (2002). Le pyothorax tuberculeux. Press Med..

[4] MagdeleinaT P, Icard PH, Pouret B (1999). Indications actuelles et résultats des décortications pulmonaires pour pleurésies purulents non tuberculeuses. Ann Chir..

[5] Mezghani S, Abdelghani A, Njima H, Hayouni A, Garrouche A, Klabi N, Benzarti M, Jerray M (2006). Le pneumothorax tuberculeux: étude rétrospective de 23 cas à l'Hôpital Farhat-Hached de Sousse, Tunisie. Rev Pneumol Clin..

[6] Woodring JH, Vandiviere HM, Fried AM (1986). The radiographic features of pulmonary tuberculosis. Am J Roentgenol..

[7] Pure kL, Licker M, Frey JG, Spiliopoulos A, Tschopp JM (2009). La fistule bronchopleurale: une complication grave de la chirurgie thoracique. Rev Med Suisse..

[8] Belmonte R, Crowe HM (1995). Pneumothorax in patients with pulmonary tuberculosis. Clin Infect Dis..

